# Precise and rapid whole-head segmentation from magnetic resonance images of older adults using deep learning

**DOI:** 10.1162/imag_a_00090

**Published:** 2024-02-22

**Authors:** Skylar E. Stolte, Aprinda Indahlastari, Jason Chen, Alejandro Albizu, Ayden Dunn, Samantha Pedersen, Kyle B. See, Adam J. Woods, Ruogu Fang

**Affiliations:** J. Crayton Pruitt Family Department of Biomedical Engineering, Herbert Wertheim College of Engineering, University of Florida, Gainesville, FL, United States; Center for Cognitive Aging and Memory, McKnight Brain Institute, University of Florida, Gainesville, FL, United States; Department of Clinical and Health Psychology, College of Public Health and Health Professions, University of Florida, Gainesville, FL, United States; Department Of Computer & Information Science & Engineering, Herbert Wertheim College of Engineering, University of Florida, Gainesville, FL, United States; Department of Neuroscience, College of Medicine, University of Florida, Gainesville, FL, United States; Department of Electrical and Computer Engineering, Herbert Wertheim College of Engineering, University of Florida, Gainesville, FL, United States

**Keywords:** whole-head segmentation, MRI, non-invasive brain stimulation, deep learning, artificial intelligence

## Abstract

Whole-head segmentation from Magnetic Resonance Images (MRI) establishes the foundation for individualized computational models using finite element method (FEM). This foundation paves the path for computer-aided solutions in fields such as non-invasive brain stimulation. Most current automatic head segmentation tools are developed using healthy young adults. Thus, they may neglect the older population that is more prone to age-related structural decline such as brain atrophy. In this work, we present a new deep learning method called GRACE, which stands for **G**eneral, **R**apid, **A**nd **C**omprehensive whole-h**E**ad tissue segmentation. GRACE is trained and validated on a novel dataset that consists of 177 manually corrected MR-derived reference segmentations that have undergone meticulous manual review. Each T1-weighted MRI volume is segmented into 11 tissue types, including white matter, grey matter, eyes, cerebrospinal fluid, air, blood vessel, cancellous bone, cortical bone, skin, fat, and muscle. To the best of our knowledge, this work contains the largest manually corrected dataset to date in terms of number of MRIs and segmented tissues. GRACE outperforms five freely available software tools and a traditional 3D U-Net on a five-tissue segmentation task. On this task, GRACE achieves an average Hausdorff Distance of 0.21, which exceeds the runner-up at an average Hausdorff Distance of 0.36. GRACE can segment a whole-head MRI in about 3 seconds, while the fastest software tool takes about 3 minutes. In summary, GRACE segments a spectrum of tissue types from older adults’ T1-MRI scans at favorable accuracy and speed. The trained GRACE model is optimized on older adult heads to enable high-precision modeling in age-related brain disorders. To support open science, the GRACE code and trained weights are made available online and open to the research community at https://github.com/lab-smile/GRACE.

## 1 Introduction

Whole-head segmentation from Magnetic Resonance Images (MRIs) establishes the foundation for individualized finite element method (FEM) (Dumont et al., 2009; Voo et al., 1996). Individual heads may vary widely in both structure and function due to age, genetic history, and other factors. Modeling the human head is highly dependent on accurate head segmentation due to differences in tissue properties. Hence, rapid, precise, and robust individualized head segmentation is necessary to capture the high irregularity, inhomogeneity, and nonlinearity of head tissue. This could largely contribute to improving patient response to therapy, reducing trial-to-trial variability, and substantially accelerating treatment planning. Therefore, accurate and robust segmentation paves the path for computer-aided intervention and treatments in fields such as non-invasive brain stimulation (NIBS) (Datta et al., 2011; Indahlastari et al., 2019), surgical simulation (Bro-Nielsen, 1998), traumatic brain injury interpretation treatment (Raul et al., 2008; Yang et al., 2014), forensics (Raul et al., 2008), connectivity analysis, and source localization in electroencephalography (EEG) and magnetoencephalography (MEG) (Cho et al., 2015). This will be particularly important to transcranial electrical stimulation (tES) and transcranial magnetic stimulation (TMS), which have high clinical potential yet suffer from heterogeneity in patient responses due to inter-individual variability (Horvath et al., 2015; Indahlastari et al., 2020).

The medical Imaging community invests significant resources into improving methods for end-to-end automated segmentation. Most publicly available segmentation tools, datasets, and challenges are typically focused on segmenting the brain instead of the entire head. Despite this, there are some key previous works in the head segmentation space that serve as useful comparisons within this work. Broadly, common head segmentation approaches can be broken down into traditional probabilistic approaches and deep learning approaches. The Realistic Volumetric Approach to Simulate Transcranial electrical stimulation (ROAST) (Y. Huang et al., 2019) segments the head tissue by combining the Statistical Parametric Mapping (SPM) toolbox (Ashburner & Friston, 2005; *SPM - Statistical Parametric Mapping*, n.d.) with custom touch-up scripts. The HEADRECO pipeline (Saturnino et al., 2019) uses the Computational Anatomy Toolbox for SPM (CAT12) (Gaser et al., 2022) to improve SPM12 segmentation. HEADRECO performs the main segmentation task within older versions of the SimNiBS pipeline (Saturnino et al., 2019). ROAST and HEADRECO are valuable tools for automatic segmentation and provide masks for semi-automatic correction. However, they do not distinguish between some key sub-tissues in NIBS research (e.g., cancellous bone versus cortical bone). Puonti et al. segment 15 tissue types in MRIs using the Complete Head Anatomy Reconstruction Method (CHARM) (Puonti et al., 2020). At present, CHARM replaces HEADRECO as the default segmentation method in SimNiBS 4.0. CHARM segments a single T1 or T2 image into 10 head tissues, including distinguishing cancellous and cortical bone. The CHARM toolbox functions based on a head atlas that is constructed from 20 young adult scans. Studies show that older adult brains are different than young adult atlases due to white matter content, grey matter content, and other factors (Indahlastari et al., 2020). The deep learning works that segment the entire head are limited due to the practical requirements of finding adequate reference segmentations. The whole-head MultiPrior Segmentation tool (MultiPrior) (Hirsch et al., 2021) combines methodologies from probabilistic methods and deep learning methods. Namely, a three-dimensional (3D) convolutional neural network (CNN) segments images using information from TPMs, morphological priors, and spatial context. Rashed et al. develop a new U-Net framework, ForkNet (Rashed et al., 2019), to segment 13 tissue types in T1 MRIs (Rashed et al., 2019). Its framework is based on a U-Net structure that combines a single CNN encoder with separate decoders that are each focused on one of the 13 tissue types. This method segments more tissue types compared to other tools. Yet, it only operates on two-dimensional (2D) MRIs. Studies that require the full volumetric MRI segmentation would need to individually input separate 2D slices for full computation.

One promising network for deep learning segmentation is the U-Net transformer (UNETR) (Hatamizadeh et al., 2021) architecture. This architecture is inspired by U-Net, but it replaces the encoder path of a traditional U-Net network with a transformer module. Transformer modules have been very successful in natural language processing (NLP) tasks due to the capability to learn long-range dependencies (Vaswani et al., 2017). Transformer modules can learn global contextual information across images solely using attention mechanisms. Networks that run on attention mechanisms have been shown to surpass networks that rely exclusively on recurrence or convolutions in terms of both performance and computational time (Vaswani et al., 2017). Indeed, recent work has shown that transformer modules can achieve impressive performance across a wide range of medical image segmentation tasks (Cao et al., 2023; Dhamija et al., 2023; Hatamizadeh et al., 2021; He et al., 2023; S. Huang et al., 2022; Karimi et al., 2022; Lee et al., 2019; Ma et al., 2022; Tang et al., 2022). UNETR pairs the success of transformers with that of U-Net-based architectures. U-Net architectures have dominated various medical image segmentations tasks since U-Net’s initial conception (Falk et al., 2019; Getao Du et al., 2020; Isensee et al., 2018; Siddique et al., 2021; *UNet++: A Nested U-Net Architecture for Medical Image Segmentation*, n.d.). Together, the advantages of U-Net and transformer modules allow UNETR to be an ideal choice for the backbone in the proposed work.

In this work, we present a new deep learning-based method called GRACE, which stands for General, Rapid, And Comprehensive whole-hEad tissue segmentation from T1-weighted structural MRIs (T1 MRIs). GRACE is trained and evaluated on a novel dataset that consists of 177 manually corrected MR-derived reference segmentations that have undergone meticulous manual review. Each T1-weighted MRI volume is segmented into 11 tissue types (white matter, grey matter, eyes, cerebrospinal fluid, air, blood vessel, cancellous bone, cortical bone, skin, fat, and muscle) that are optimal for computational head modeling in NIBS pipelines. The motivation of this paper is to provide a fully automatic segmentation tool that is optimal for the older adult population, who are the main treatment group in cognitive aging and dementia studies. The current GRACE model can be used as part of a larger head modeling pipeline for the best overall performance. This work supports that GRACE can be adjusted to different numbers of tissue types (5 or 11 tissues) so that it can be fit to different tasks and existing head modeling tools. These results can contribute to any application of volume conductor models for studies involving older adults. In all, GRACE is an important step in improving the status and effectiveness of head modeling tools for precision treatment in older adults.

## 2 Materials and Methods

GRACE is trained and validated using a total of 177 T1 MRI data from a healthy older adult cohort (mean age: 73 years, std: 5 years) split into 137 for training, 20 for validation, and 20 for testing. The same testing data are used for all comparisons to other software. Trained research staff derives the reference segmentations using automatic segmentation followed by manual correction (i.e., semi-automated segmentation) with a reference of an atlas (Spitzer & Whitlock, 1998). After training, the final GRACE model segments unseen MRIs into 11 tissue types, namely white matter (WM), grey matter (GM), eyes, cerebrospinal fluid (CSF), air, major artery (Blood), cancellous bone, cortical bone, skin, fat, and muscle. The entire pipeline is described in the subsections below.

### 2.1 Dataset and image scanning parameters

This study harnesses data from the Augmenting Cognitive Training in Older Adults (ACT) trial (NCT02851511). The ACT trial is a Phase III randomized clinical trial that tests the effectiveness of cognitive training paired with transcranial direct current stimulation (tDCS) for cognitive improvement (Woods et al., 2018). This study includes 379 participants at the University of Florida (Gainesville, FL, USA) and the University of Arizona (Tucson, AZ, USA). The participants of the study are cognitively healthy older adults within the age range of 65 to 89 years. Exclusion criteria include neurological disorders, cognitive impairment, opportunistic brain infection, major psychiatric illness, unstable or chronic medical conditions, MRI contraindications, physical impairment precluding motor response, GABA-ergic medications, or left-handedness. The Institutional Review Boards (IRBs) of both institutions approved the study protocol. The study staff obtained informed written consent from all participants. GRACE uses segmentation data from the T1-MRIs of 177 ACT study participants since this was what had been completed for reference segmentations at the time of this study. These 177 participants include data from 107 female participants and 70 male participants. Most of these participants are racially white (157/177).

MRI imaging parameters are as follows: Structural T1-weighted magnetic resonance images (T1-MRIs) are obtained using a 3-Tesla Siemens Magnetom Prisma scanner with a 64-channel head coil at the University of Florida (UF) and a 3-Tesla Siemens Magnetom Skyra scanner with a 32-channel head coil at the University of Arizona. The participants are given earplugs to reduce the harmful effects of scanner noise. Foam padding is used to reduce participant head motion. The scanning parameters included a repetition time (TR) = 1800 ms, echo time (TE) = 2.26 ms, resolution = 1.0 × 1.0 × 1.0 mm^3^, and Field-of-view (FOV) = 256 × 256 × 176 mm. Among the 177 research participants, 113 participants came from the UF study site and 64 came from the AZ study site. The average Signal-to-Noise ratio (SNR) of this dataset is 12.1 ± 1.54.

### 2.2 Reference segmentations

**Fig. 1.**
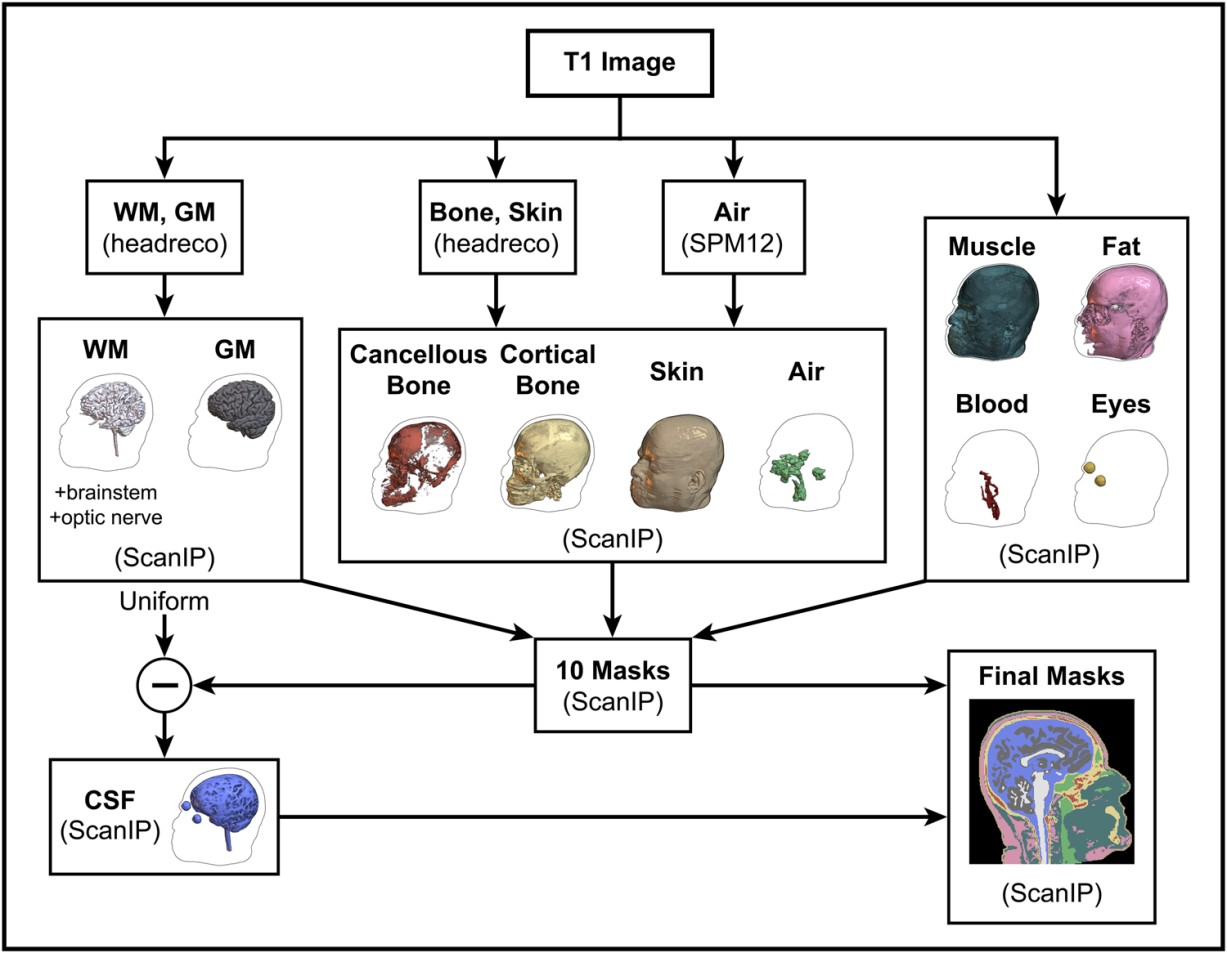
The overall semi-automated segmentation pipeline for the reference segmentations. The T1 Image (“uppermost box”) represents the starting image. Each following box represents either incomplete tissue masks (tissue names in bold) or finished masks (“masks” in bold). The methods used to compute the mask are in parentheses in the corresponding box.

**Fig. 2.**
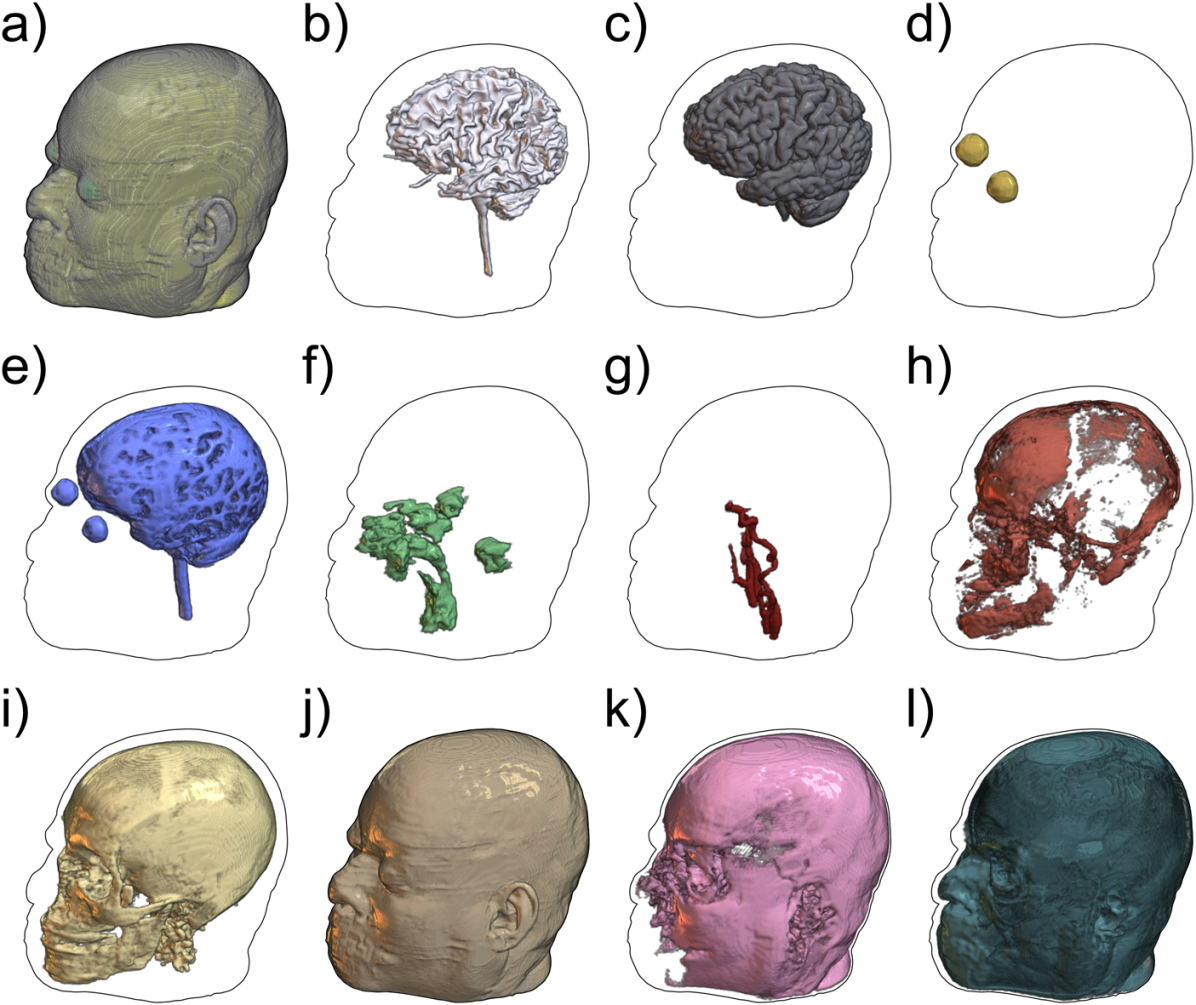
3D rendering of the 11 tissue masks as visualized in MRIcroGL (Rorden & Brett, 2000). Each tissue is represented in the same color label that is selected for that tissue in Figure 3. The order of the binary masks is as follows: (a) full head rendering, (b) WM, (c) GM, (d) eyes, (e) CSF, (f) air, (g) blood, (h) cancellous bone, (i) cortical bone, (j) skin, (k) fat, (l) muscle.

**Fig. 3.**
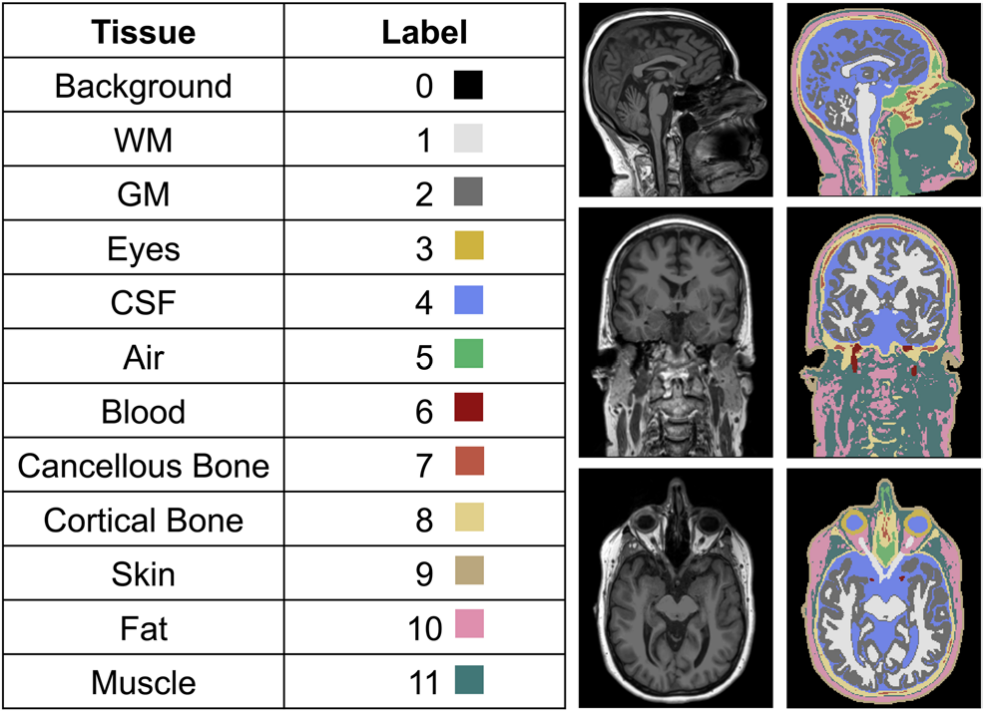
The chart on the left depicts the names of each of our 11 tissue types and the corresponding labels (number and color). The two columns on the right show an example T1 MR image (left column) and corresponding ground truth segmentation (right column). The top row is the sagittal plane, the second row is the coronal plane, and the last row is the axial plane.

A trained staff of four dedicated manual annotators, referred to as segmentors in this paper, segmented the research participants’ T1 MRIs into 11 tissue types using a semi-automated labeling procedure. These 11 tissues were selected to best serve tDCS modeling (Indahlastari et al., 2016). For this process, the team applied the methods described by Indahlastari et al. (2016) with some modifications to further improve the segmentation results, as shown in Figure 1. All automatic segmentation outputs were manually corrected in the ScanIP module in Simpleware™ software version 2018.12 (Synopsys, Inc., Mountain View, USA). Base segmentations for WM, GM, and bone were obtained using HEADRECO, while the air compartment was generated in SPM12. HEADRECO’s segmentations were based on SPM12, but it was also run with CAT12 to refine the results. CAT12 greatly improved the base WM, GM, and bone segmentations, but the air segmentation was qualitatively less accurate than the base SPM12. The brainstem, spinal cord, and optic nerves were manually segmented from the T1 and combined with the WM mask. The bone compartment was further classified into cancellous and cortical tissue using thresholding and morphological operation in Simpleware. The major artery visible on T1 images (labeled as blood in this work), skin, fat, muscle, and eyes (sclera and lens) were also manually segmented in Simpleware. CSF was generated by subtracting the final 10 tissue types from the entire head volume. The final 11 tissue masks served as the segmentation labels for training the GRACE algorithm. The remainder of this paper refers to the combined 11-tissue masks as “reference segmentations.” These reference segmentations serve as the point of comparison for the outputs of different head segmentation approaches. Figure 2 shows the three-dimensional (3D) visualizations of each of the 11 tissues in greater detail. Figure 3 shows the labels that correspond to each tissue following a similar color scheme as CHARM (Puonti et al., 2020). Note that the blood segmentation is limited to the extent that blood is visible in T1-MRI images. This is because GRACE does not rely on additional imaging modalities to acquire its reference segmentations or to predict the head segmentations of MRIs.

### 2.3 Data preprocessing

#### 2.3.1 Preprocessing pipeline

All preprocessing procedures are performed in Medical Open Network for Artificial Intelligence (MONAI) (*MONAI - Home*, n.d.). All information in this section refers to steps that are taken for both GRACE and U-Net. All preprocessing is consistent between the two algorithms.

#### 2.3.2 Data (training/validation/testing) preparation

The raw T1-MRIs are normalized such that all voxel values ranged between 0 and 1 in double-precision floating-point format. All images and labels are converted into tensors. No other pre-processing steps are required at inference time.

#### 2.3.3 Training data augmentation

**Fig. 4.**
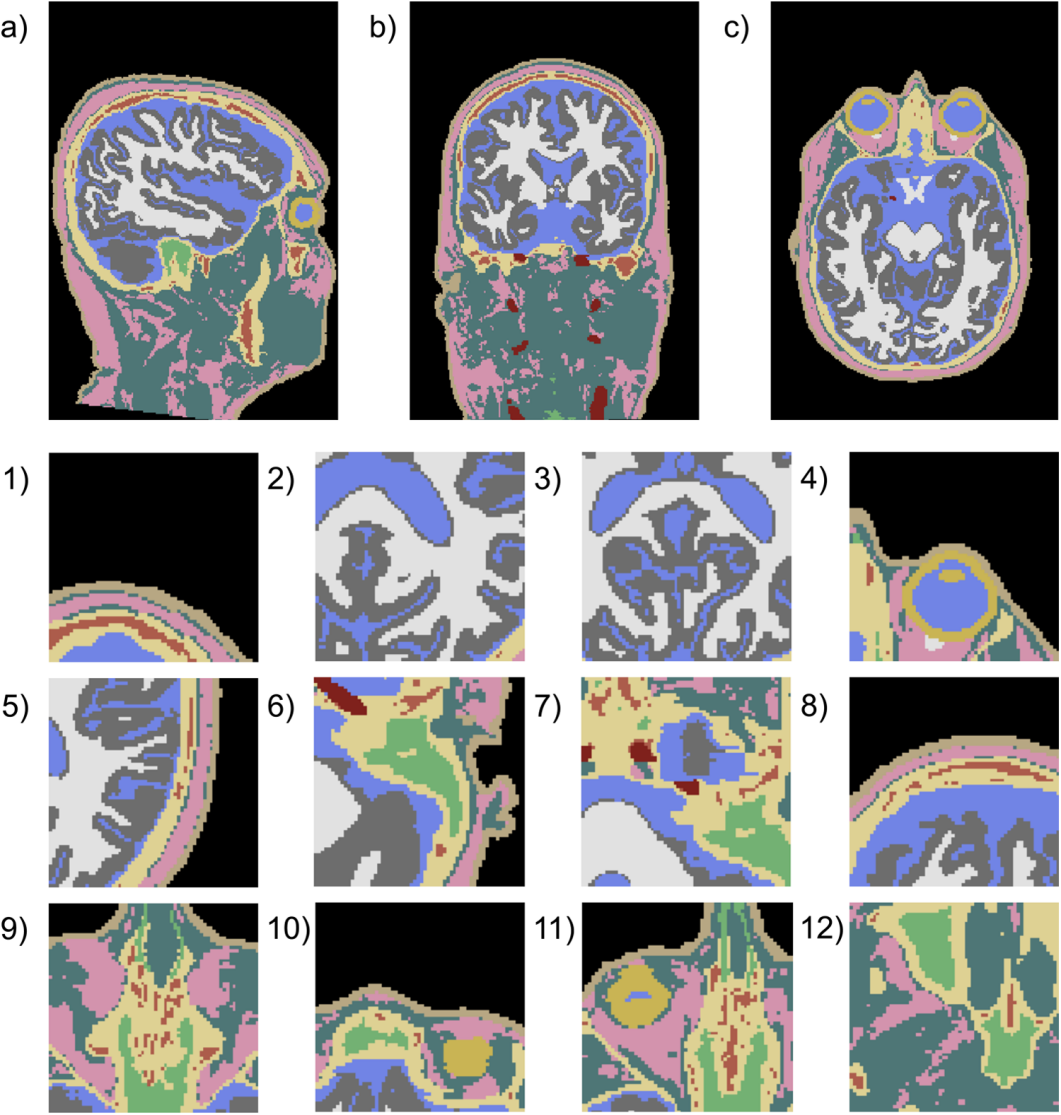
The training data loader generates 12 data samples of size 64 × 64 × 64 per T1-MRI input. Each data label is the center pixel at least once. (a) – (c) represent the sagittal, coronal, and axial views of an original T1-MRI volume of size 256 × 256 × 176. The patches have each of the following labels as its center pixel: (1) Background, (2) WM, (3) GM, (4) Eyes, (5) CSF, (6) Air, (7) Blood, (8) Cancellous Bone, (9) Cortical Bone, (10) Skin, (11) Fat, and (12) Muscle.

To improve model performance, a preliminary phase augments the training data by cropping each 256 × 256 × 176 head volume into 12 smaller 3-dimensional patches of 64 × 64 × 64 voxels. The cropping process randomly selects these 12 patches such that each of the 12 labels in Figure 3 (11 tissues + background) constitutes the center pixel of one patch, as shown in Figure 4. Data augmentation is also performed by flipping the training volumes horizontally or vertically with a probability of 0.1. In addition, the data loader randomly adds Gaussian noise (mean = 0, standard deviation = 0.1) to training images with a probability of 0.1. This data augmentation process makes the GRACE model and the comparison U-Net more robust to data variability due to different scanners, settings, or sequences, as well as noise from the image acquisition process. The above data augmentation is applied to the training data only, rather than also including the validation or testing data, to ensure the rigor of the evaluation process.

### 2.4 U-Net transformer (UNETR) architecture

**Fig. 5.**
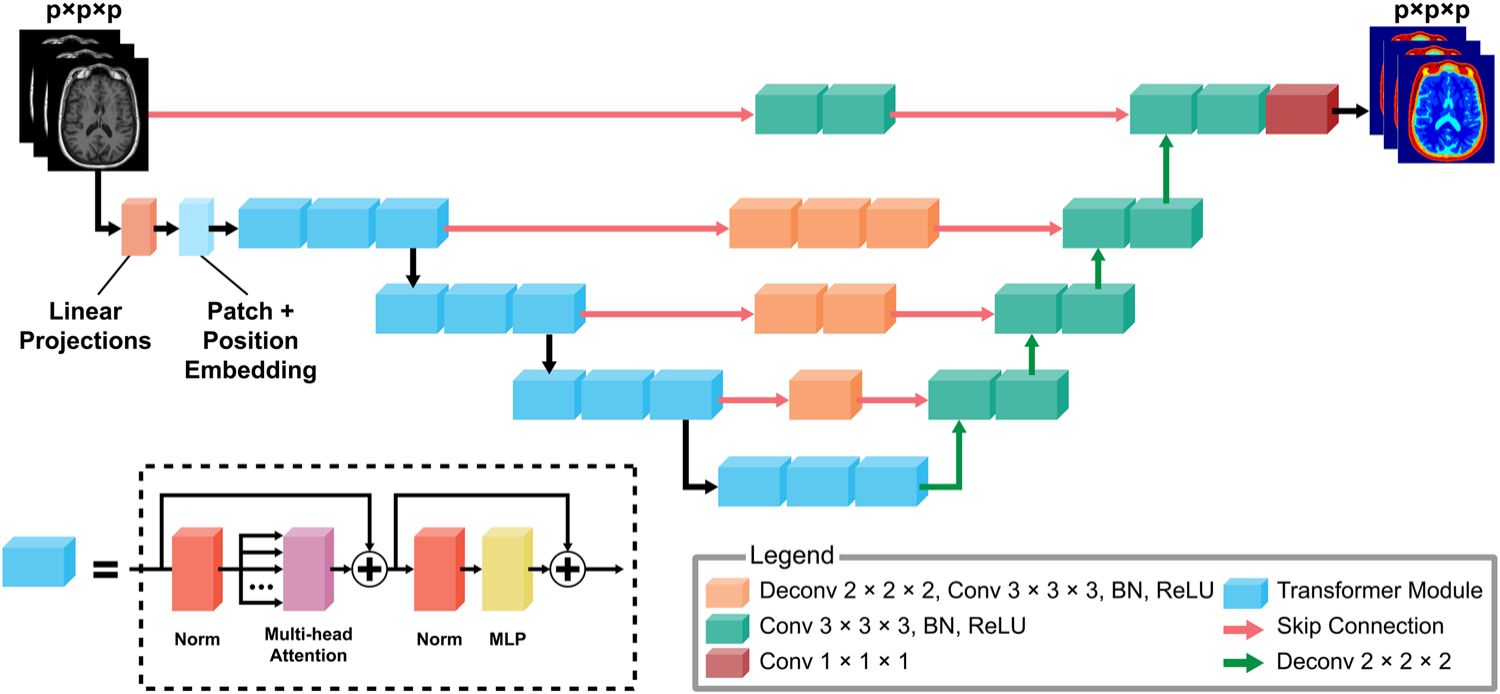
The UNETR architecture. The architecture inputs an image subsample of size p×p×p (p = 64 in this paper) from whole-head image(s) (of size 256 × 256 × 176). The transformer encoder and fully convolutional decoder are connected by skip connections (pink arrows).

GRACE uses the U-Net transformer (UNETR) (Hatamizadeh et al., 2021) architecture which replaces the encoder path of a traditional U-Net network with a transformer module. Transformer modules have been very successful in natural language processing (NLP) tasks due to the capability to learn long-range dependencies (Vaswani et al., 2017). UNETR inputs 3D imaging data as individual 1D sequences from patch-wise image inputs. The transformer encoder learns the key information and relationships within and between patch items in the “sequence” using attention-based learning. Attention-based learning focuses on the high resolution of important focal points in the input image, whereas less important areas of the image are at low resolution. Transformers overcome vanishing gradient issues in long-range sequences through multi-head attention layers. Multi-head attention layers learn more global contextual information than a traditional fully convolutional network (FCN)-based encoder. UNETR uses an FCN-based decoder as is also commonly implemented in the standard 3D U-Net. Skip connections link the transformer-based encoder and the FCN-based decoder. Figure 5 shows the architecture of UNETR.

### 2.5 Comparison algorithms

The traditional 3D U-Net architecture serves as an additional comparison network for GRACE. This model is trained to classify all 11 tissues (12 output channels). The 3D U-Net up-samples the image input to obtain feature maps of sizes (32, 32, 64, 128, 256, 32) and employed dropout with a 0.5 probability. This study uses the MONAI version of 3D U-Net (Falk et al., 2019). Furthermore, GRACE is compared to the freely available software tools SPM12, HEADRECO, CHARM, ForkNet, and MultiPrior.

### 2.6 Evaluation metrics

#### 2.6.1 Dice score

Dice (1945) represents the overlap of two binary masks:

$$Dice=\frac{2\left|Y\cap \hat{Y}\right|}{\left|Y\right|+\left|\hat{Y}\right|}$$

where Y and $\hat{Y}$ represent the ground truth mask and the generated mask for a given tissue, respectively. This means that we compute the Dice score for each tissue individually. A mask for a given tissue is an image/volume matching the original image/volume size which only contains 1’s (given tissue type present) and 0’s (given tissue type absent). A perfect overlap between these two binary masks generates a Dice score of 1, whereas a 0 represents no mask overlap.

#### 2.6.2 Average Hausdorff distance

Huttenlocher et al. (1993) calculates the average of the maximum distances between the closest points in two data subsets. It is in units of mm.

$$H(Y,\;\hat{Y})=\text{mean}\left(h(Y,\hat{Y}),\;h(\hat{Y},Y)\right)$$

$$h(Y,\hat{Y})=\underset{y\;in\;Y}{\max}\left(\underset{\hat{y}\;in\;\hat{Y}}{\min}(d(y,\hat{y})\right)$$

$$h(\hat{Y},Y)=\underset{\hat{y}\;in\;\hat{Y}}{\max}\left(\underset{y\;in\;Y}{\min}(d(\hat{y},y)\right)$$

where Y is the ground truth mask for a given tissue, $\hat{Y}$ is the generated mask for a given tissue, *y* represents a pixel in *Y*, and $\hat{y}$ represents a pixel in $\hat{Y}$. $H(Y,\hat{Y})$ is the overall Hausdorff Distance, whereas $h(Y,\hat{Y})$ and $h(\hat{Y},Y)$ are directed Hausdorff Distances. Each directed Hausdorff Distance measures the maximum distance between the closest points in the ground truth and generated masks. The distance measures are denoted as $d(y,\hat{y})$ and $d(\hat{y},y)$, which are Euclidean distances. The average Hausdorff Distance takes the average of the directed Hausdorff Distances. Smaller Hausdorff Distances indicate better segmentation. The remainder of this work refers to average Hausdorff Distance as Hausdorff Distance.

### 2.7 Tissue aggregation

**Fig. 6.**
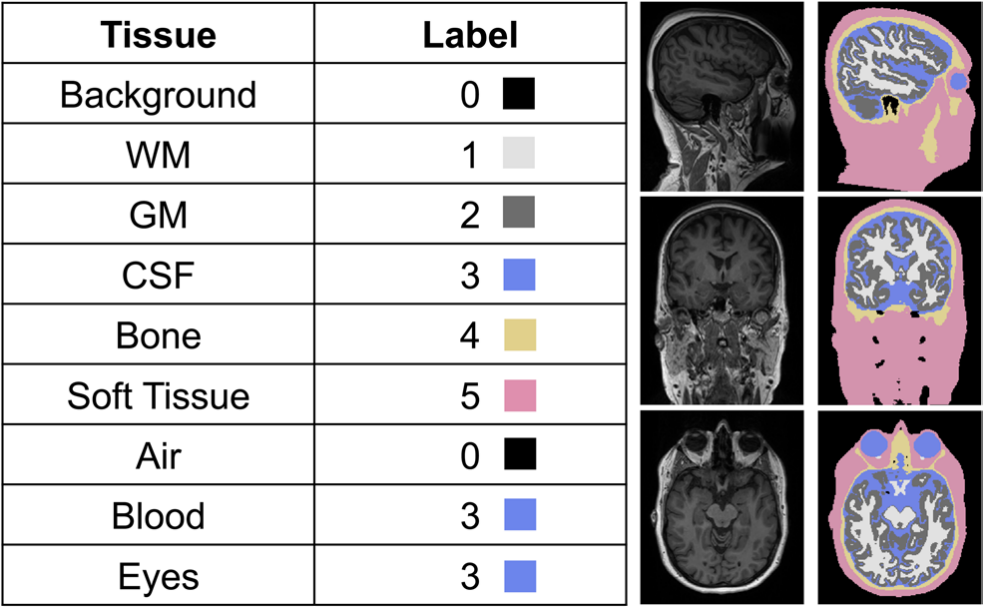
The left table shows the condensed tissue classes and their corresponding number and color labels. Blood is zeroed out due to it not smoothly falling into one of these broader tissue categories. The two columns on the right show an example T1 MR image (left column) and corresponding ground truth segmentation (right column). The top row is the sagittal plane, the second row is the coronal plane, and the last row is the axial plane.

The final tissue masks from each method are combined into larger class groupings for comparison purposes, as the different methods provide different tissue labels. Table 1 is the tissue conversion chart that is used in this aggregation. This scheme is chosen so that the comparisons can be as fair as possible. Figure 6 depicts the condensed tissue classes in pictorial form. GRACE and U-Net are not re-trained on five tissues; the tissue masks are combined accordingly.

**Table 1.** Table showing which labels from each method are aggregated into combined labels for comparison purposes.

Combined tissue name	CHARM	SPM and MultiPrior	HEADRECO	ForkNet	GRACE and U-Net	Combined label
Background (BG)	BG	BG	BG	BG	BG	0
WM	WM	WM	WM	WM	WM	1
GM	GM	GM	GM	GM	GM	2
Eyes^a^	Eyeballs	N/A	Eyes	Vitreous Humor	Eyes	3
CSF^a^	CSF	CSF	CSF, Ventricles	CSF	CSF	3
Bone	Compact bone, Spongy bone	Bone	Bone	Cancellous bone, Cortical bone	Cancellous bone, Cortical bone	4
Soft tissue	Scalp, Muscle	Soft Tissue	Soft Tissue	Muscle, Fat	Skin, Fat, Muscle	5
Blood^a^	Blood	N/A	N/A	Blood	Blood	3
Air	BG	Air, Sinus Cavities	Air	Mucous	Air	0
Cerebellum	N/A	N/A	N/A	Cerebellum	N/A	6^b^
Dura	N/A	N/A	N/A	Dura	N/A	3

a *The* *eye and blood regions in the reference segmentations are zeroed out for the 5-tissue comparisons. These masks are omitted only for segmentation evaluation purposes and are not indicative of our final masks in current flow models.* The exclusion is because GRACE and CHARM are different in their definitions of the eye and blood labels to complete a fair comparison. These sections are excluded by masking out (i.e., setting to zero values) the positive coordinates in the reference eye mask from all algorithms’ output masks. The eye and blood segmentations are still shown visually to display the strengths and weaknesses of each algorithm’s full segmentation capacity. The areas that the reference segmentations identify as CSF within the eyes are also masked out from the CSF mask for the fairest CSF comparison. In addition, the CHARM blood regions are grouped with its CSF mask. This is because the reference segmentations label venous structures as occurring within CSF due to the limitations of T1-only annotations.

b Cerebellum is zeroed out from the reference segmentation and ForkNet for the ForkNet comparison only.

### 2.8 Qualitative study on improving the semi-automated segmentation using human-computer interaction

The final experimental results consist of qualitative analysis concerning the potential for GRACE to help improve the semi-automated segmentation of the reference segmentations. The reference segmentations that are used for comparison in this paper are not influenced by GRACE segmentation results. However, the manual segmentors in this study examine GRACE’s potential for use in a human-computer interactive manner in future works. These procedures are purely qualitative at this time, but future work will extend this experimental section with statistical findings. This study is important because it further demonstrates GRACE’s advantage of accurate automatic segmentations from only T1-MRI inputs. Certain tissue types have an upper limit on segmentation accuracy without referencing other head imaging modalities (e.g., angiogram of T2-weighted images). In this case, the manual segmentors particularly focus on GRACE’s potential to improve blood segmentations using only T1-MRIs.

### 2.9 Segmentor consistency

It is important to evaluate the consistency of tissue segmentation quality across segmentors to validate GRACE’s reference segmentations. Specifically, consistency is assessed by calculating the Dice score in each tissue across the group of segmentors.

The Dice score is first calculated in pairs of segmentors for each tissue type (i.e., white matter, gray matter, CSF, bone, muscle, fat, skin, air, eyes, blood vessels, and uniform). While the uniform mask is not modeled, its construction is critical for segmenting the eyes, fat, and muscle masks. The first step of the calculation is to compute individual Dice score for each segmentor pair. Then, the combined ratings are calculated by averaging the Dice scores across the group of segmentors. The Dice scores are computed in practice head models. During the process of segmenting participant data, three annotators serve as the three stages of quality control to ensure the final segmentation product in each head is consistent across participants and meet the established protocols.

## 3 Implementation

### 3.1 GRACE and U-Net implementations

GRACE and 3D U-Net are both implemented using the Medical Open Network for Artificial Intelligence (MONAI) (*MONAI - Home*, n.d.), which is an open framework for medical imaging written in PyTorch. The total dataset includes 177 T1-MRIs split into 137 for training, 20 for validation, and 20 for testing. Each 256 × 256 × 176 head volume is sampled into a total of 12 patches of size 64 × 64 × 64. GRACE processes the 64 × 64 × 64 patch inputs as sequences of 64 16 × 16 × 16 non-overlapping patches, whereas U-Net inputs the full 64 × 64 × 64 patch inputs. Both models use the same training/validation/testing split. Both models use a training batch size of 10 image volumes and a validation size of 10 image volumes, where the patch sampling process results in a training batch size of 120 image volumes. Both models have randomly initiated weights for all network layers. GRACE and U-Net both use a loss function that is a weighted sum of Dice and cross-entropy loss (DiceCE) (Taghanaki et al., 2021). The loss includes the background label, as the algorithm needs to detect the head location in the image. An Adam optimizer updates each model’s parameters with a learning rate of 10-4 and weight decay of 10-5. GRACE and U-Net each train for 2500 epochs with validation at every 50 epochs. The final model for each method is selected based on the best overall performance during validation. Hence, the traditional U-Net uses the same parameters and the same number of epochs for comparison purposes.

### 3.2 Network training and inference

The GRACE and U-Net each train on one A100 NVIDIA graphics processing unit (GPU) on the University of Florida (UF)’s supercomputer HiPerGator (*HiPerGator—Research Computing—University of Florida*, n.d.). This training also requires 4 central processing units (CPUs) and 30 GB of random-access memory (RAM). Training with these parameters takes an average of 27 hours. On these same resources, a trained model segments a new head volume in about 3 seconds of inference time.

## 4 Results

### 4.1 Quantitative results on the 11-tissue segmentation task

**Fig. 7.**
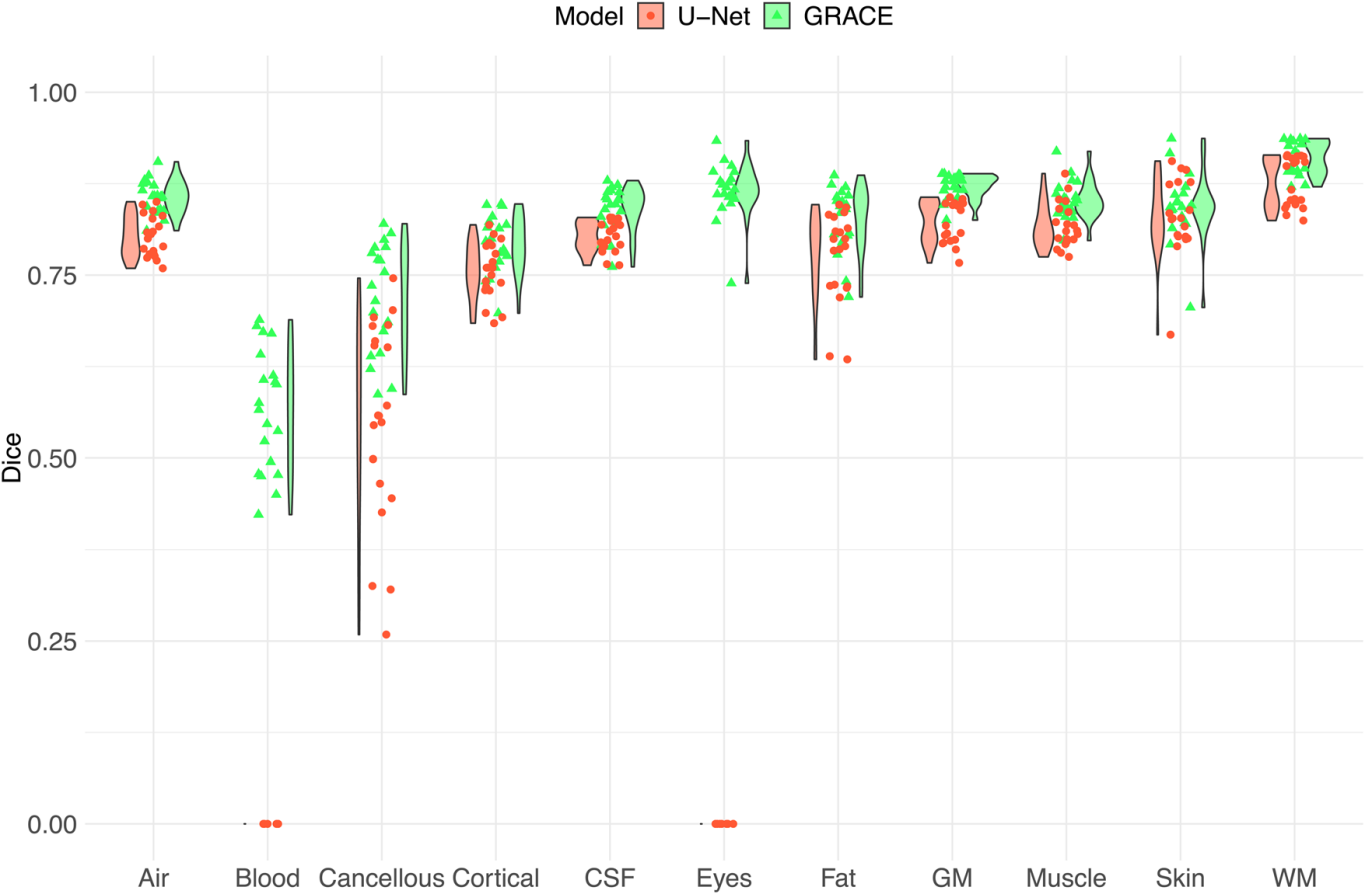
Dice scores of GRACE on the 11-tissue segmentation task as compared to the traditional 3D U-Net architecture. Dice score ranges from a minimum of 0 (worst score) to a maximum of 1 (best score). Box plots representing the interquartile range for each method per tissue. Each “dot” represents a method’s performance per tissue per individual testing MRI volume.

**Fig. 8.**
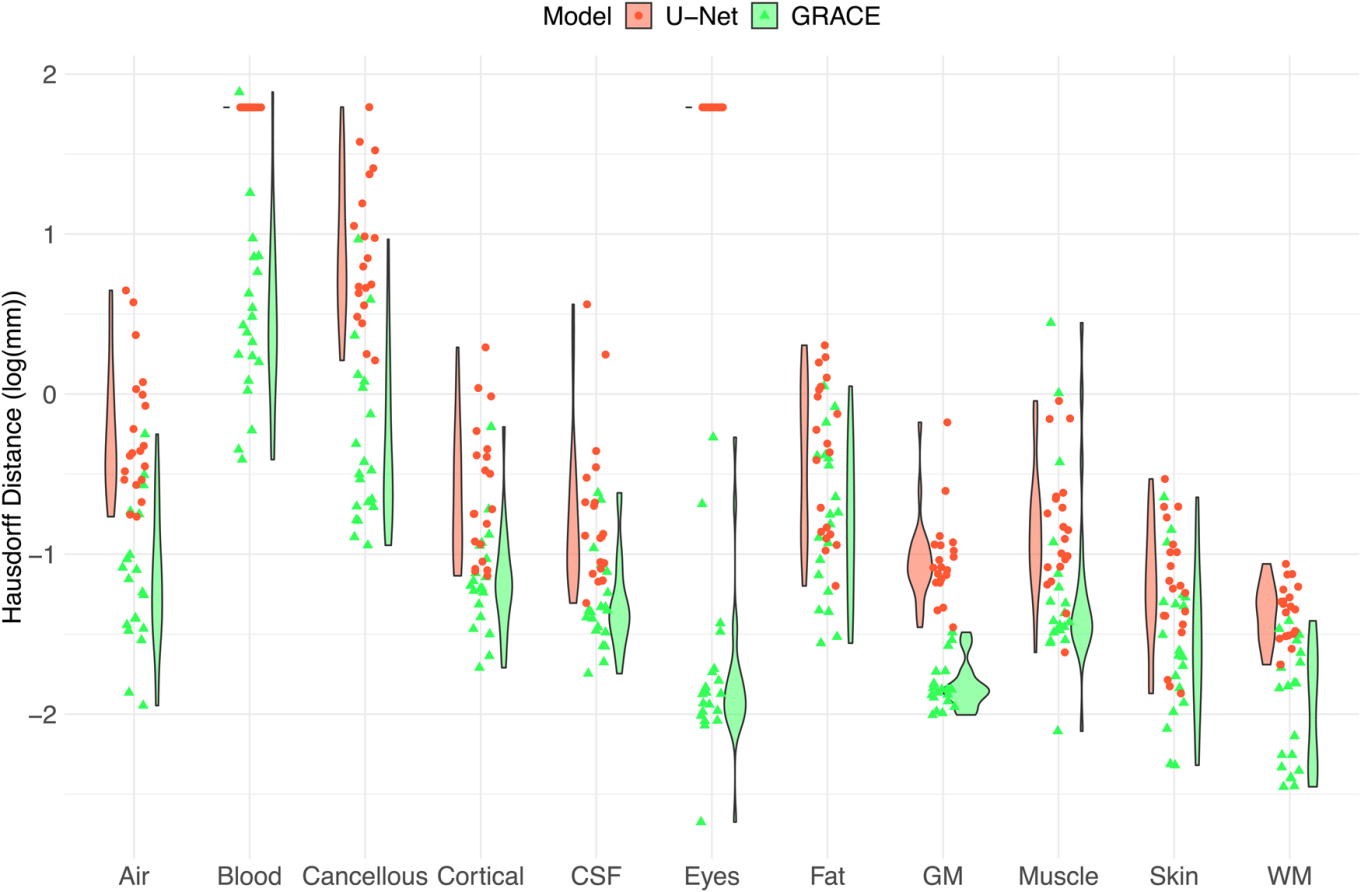
Hausdorff Distances of GRACE on the 11-tissue segmentation task as compared to the traditional 3D U-Net architecture. The best theoretical Hausdorff Distance is 0, which indicates perfect overlap. A Hausdorff of 0 would produce the most negative result possible on the natural logarithm scale. Box plots representing the interquartile range for each method per tissue. Each “dot” represents a method’s performance per tissue per individual testing MRI volume.

This section depicts the quantitative results for the 11-tissue segmentation task. This section only compares GRACE to the traditional 3D U-Net architecture because no public head segmentation tools use the exact same tissue types as GRACE. Hence, future sections compare as many overlapping tissues as possible, whereas this section solely focuses on deep learning methods. Table 2 summarizes the main findings of this experiment. These average Dice and Hausdorff Distances indicate that GRACE is superior to 3D U-Net. Figure 7 breaks the Dice scores down into each of the 11 tissue types. This figure shows that GRACE is roughly equal or better than U-Net across tissues. The most telling feature of this figure is the performance on the eye and blood masks. These tissues are not captured well by U-Net at all – this discrepancy appears to be the biggest contributor to the difference in performance. Interestingly, eye and blood were the two tissues that had the lowest total number of voxels in the T1 MRI scans. Table 3 shows the results of Wilcoxon signed-rank tests (Woolson, 2008) for GRACE versus U-Net across individual tissue types. This paired test shows that the hypothesis that the subtraction of the paired samples between the two algorithms comes from a distribution of zero median can be rejected at the 1% p-value for 10 out of 11 tissues. Figure 8 separates the tissue types based on the natural logarithm of the Hausdorff Distances. Figure 8 follows the same trend as Figure 7; namely, GRACE is equal or better than U-Net across all tissues, whereas U-Net cannot capture eye or blood at all. Note that U-Net’s scores on these two tissues are depicted as horizontal lines on these features. The horizontal lines occur at the worst score possible for the given evaluation metric. Specifically, Figure 7 depicts U-Net’s Dice scores for eyes and blood as single points at 0. Figure 8 shows similar point-wise performance for U-Net’s Hausdorff Distances just below 2. Table 4 performs the Wilcoxon signed-rank test in respect to Hausdorff Distance. The hypothesis that the subtraction of the paired Hausdorff Distances comes from a distribution of zero median can be rejected for every tissue type. This means that GRACE is statistically better than U-Net in the Hausdorff Distances for all 11 tissues.

**Table 2.** This table summarizes the average metrics for GRACE and 3D U-Net on 11 tissue types.

Method	Average dice **↑**	Average Hausdorff distance **↓**
U-Net	0.64	4.63
*GRACE*	**0.82**	**2.87**

An ideal Dice score is 1.0 and the worst Dice score is 0.0, which means that higher Dice scores are better (arrow pointing up). The ideal Hausdorff Distance is 0.0, such that lower Hausdorff Distances are better (arrow pointing down). The bold values indicate the best-performing model for the given metric.

**Table 3.** Results of a paired test (signed-rank) for determining if the tissue outputs for GRACE and U-Net are statistically different in Dice score.

Tissue	$\begin{array}{l} \mu_{GRACE}-\\ \;\mu_{U-Net} \end{array}$	Signed-rank P-value	Statistically significant?
WM	0.034	8.86e-05	Yes
GM	0.049	8.86e-05	Yes
Eyes	0.866	8.86e-05	Yes
CSF	0.039	1.20e-04	Yes
Air	0.054	8.86e-05	Yes
Blood	0.566	8.86e-05	Yes
Cancellous Bone	0.168	8.86e-05	Yes
Cortical Bone	0.035	8.86e-05	Yes
Skin	0.015	1.52e-02	No
Fat	0.051	1.03e-04	Yes
Muscle	0.035	8.86e-05	Yes

These results show that GRACE is statistically better than U-Net in 10 of the 11 tissues.

**Table 4.** Results of a paired test (signed-rank) for determining if the tissue outputs for GRACE and U-Net are statistically different in Hausdorff Distance.

Tissue	$\begin{array}{l} \mu_{GRACE}-\\ \;\mu_{U-Net} \end{array}$	Signed-rank P-value	Statistically significant?
WM	-0.108	8.86e-05	Yes
GM	-0.207	8.86e-05	Yes
Eyes	-5.799	8.86e-05	Yes
CSF	-0.251	1.63e-04	Yes
Air	-0.516	8.86e-05	Yes
Blood	-4.136	1.03e-04	Yes
Cancellous Bone	-1.890	1.03e-04	Yes
Cortical Bone	-0.258	4.49e-04	Yes
Skin	-0.088	6.42e-03	Yes
Fat	-0.245	5.11e-03	Yes
Muscle	-0.095	8.03e-03	Yes

These results show that GRACE is statistically better than U-Net in 11 of the 11 tissues. Note that unlike Figure 8, these results are on the mm scale (not the log(mm) scale).

### 4.2 Quantitative results on 5-tissue segmentation task

In this task, the same 20 testing MRI volumes from the previous section are used for testing; however, tissues are combined into larger label classes for comparison purposes. Table 5 summarizes the average Dice and Hausdorff Distances across all combined tissue types for each of the comparison methods. These results show that GRACE achieves the highest overall Dice and lowest overall Hausdorff Distance. This means that GRACE performs better than each of the other methods on average, even when only limited to the tissue types that are available from all methods. Note that eyes and blood are not included in this comparison since their definitions between software are too inconsistent. This means that the CSF scores do not include the eye portion. CHARM’s quantitative metrics approximate CHARM’s definition of blood as CSF to be more in agreement with our T1-derived reference segmentations.

**Table 5.** This table summarizes the average metrics for each method across the five condensed tissue types.

Method	Average dice **↑**	Average Hausdorff distance **↓**
ForkNet	0.58	2.45
CHARM	0.74	0.72
SPM	0.78	2.57
MultiPrior	0.84	0.52
U-Net	0.86	0.36
HEADRECO	0.85	0.41
GRACE	**0.89**	**0.21**

An ideal Dice score is 1.0 and the worst Dice score is 0.0, which means that higher Dice scores are better (arrow pointing up). The ideal Hausdorff Distance is 0.0, such that lower Hausdorff Distances are better (arrow pointing down). The bold values indicate the best-performing model for the given metric.

**Fig. 9.**
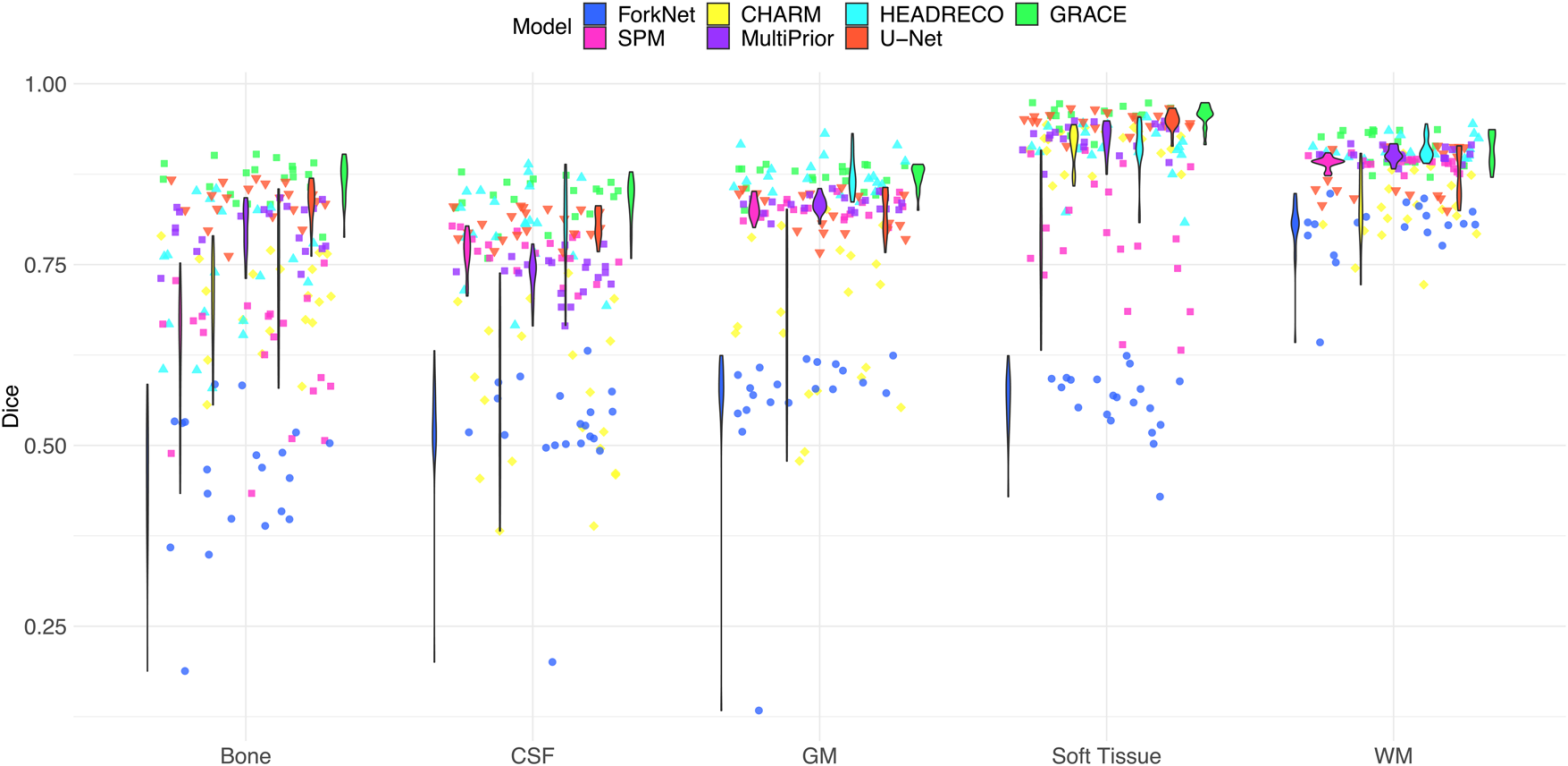
Dice score of GRACE as compared to six different methods that are common for head segmentation on a 5-tissue segmentation task. The results are shown for five tissues to fairly compare across methods with different tissue outputs. Dice score ranges from a minimum of 0 (worst score) to a maximum of 1 (best score). Box plots representing the interquartile range for each method per tissue. Each “dot” represents a method’s performance per tissue per individual testing MRI volume.

Figure 9 depicts the Dice scores for GRACE as compared to six other popular methods for head segmentation. This figure displays the specific Dice scores for each tissue type across all the comparison methods. These results demonstrate that GRACE achieves the highest Dice scores for CSF, bone, and soft tissue. GRACE obtains comparable Dice scores for WM and GM to HEADRECO.

**Fig. 10.**
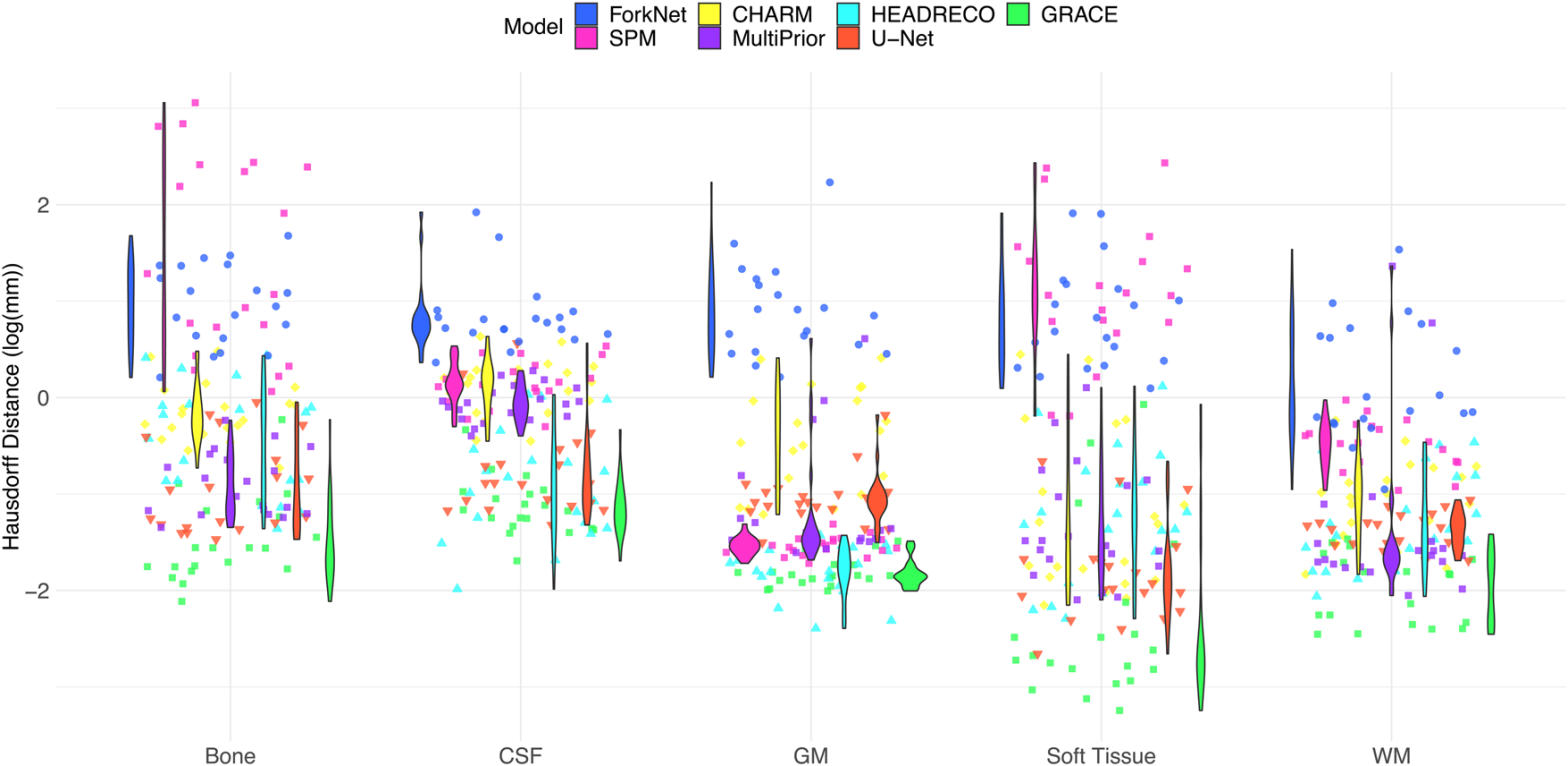
Comparison of the Hausdorff Distances using GRACE and six different methods that are common for head segmentation. The results are shown for five tissues to fairly compare across methods with different tissue outputs. The best theoretical Hausdorff Distance is 0, which indicates perfect overlap. A Hausdorff of 0 would produce the most negative result possible on the natural logarithm scale. Box plots representing the interquartile range for each method per tissue. Each “dot” represents a method’s performance per tissue per individual testing MRI volume.

Figure 10 features the corresponding results for the Hausdorff Distance metric. GRACE scores the best (the lowest) in its Hausdorff Distance for CSF, Bone, and Soft Tissue, and GRACE is comparable to HEADRECO in WM and GM.

### 4.3 Qualitative results

**Fig. 11.**
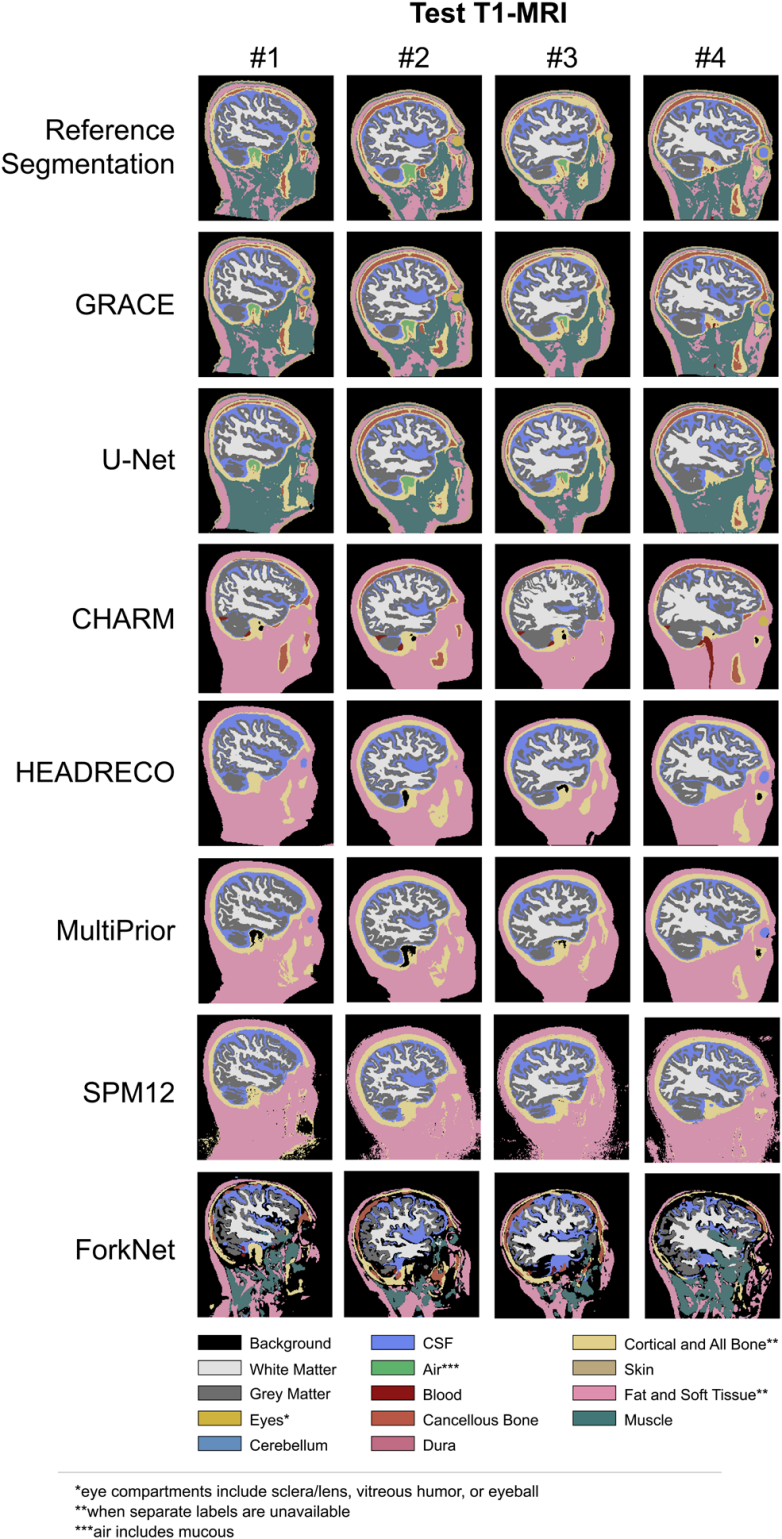
Sample segmentations from the T1-MRIs of four of the study test participants. The results are shown in the Coronal view. Column – participants; Row – segmentation models.

Figure 11 displays the coronal slices from four different research participants in the test dataset. The segmentation results roughly capture the head details for the most part; however, the SPM12 results show a consistent issue concerning soft tissue voxels being placed outside of the head. The skin boundaries in Test T1-MRI #2 - #4 are noisy in the SPM12 results. These images have background voxels placed within the soft tissue segmentations. In addition, the SPM12 segmentation for Test T1-MRI #1 places the background within the mouth area. It can also be noted that CHARM initially produced poorly registered results on Test T1-MRI #1. This research participant needed to be run through CHARM twice to fix the affine registration to obtain the result in Figure 11. This process is fixable; however, it doubles the time needed for segmentation. Also, CHARM misses a large degree of CSF in the skull cavity across all four testing examples. This can be observed by comparing the presence of the purple-colored tissue in the CHARM skull cavity to the other methodologies. CHARM segments the eyes well when they are present. CHARM’s segmentation of blood in Test T1-MRI #4 may actually be anatomically correct, as the reference segmentations in this paper only use what is available when manually segmenting from T1 MRI. HEADRECO captures the head shape and brain matter, the best among the freely available software tools. It is particularly strong at handling the WM and GM segmentations, whereas it struggles the most with bone. HEADRECO results on Test T1-MRI #1 underestimate bone in the back of the head and directly behind the eyes. The HEADRECO segmentation in Test T1-MRI #3 overestimates the bone structure and places it in contact with the background (i.e., there is an area of bone with no soft tissue in between it and the background). The MultiPrior tool segments these participants’ heads somewhat similarly to HEADRECO; however, it misses some key details. Some example areas where MultiPrior has issues include the eyes and jaw, which are both segmented as “thinner” structures. In other terms, the presence of these tissues is detected correctly, but a large portion of their pixels are incorrectly labeled as the surrounding tissue. CSF is also incorrectly labeled as GM in the back of the head. SPM12, CHARM, and HEADRECO are all generally not able to distinguish (internal) air with high consistency. Further, the shape of the head in HEADRECO’s output for Test T1-MRI #3 is misshapen in the front of the face. HEADRECO also misses the eyes in Test T1-MRI #2 and #3. U-Net yields somewhat similar results to GRACE; however, notable details are lacking in its segmentation results. U-Net misses cancellous bone in the jaw across all four testing examples. Test T1-MRI #2 and #3 are also missing eye structures in the U-Net segmentation. Also, U-Net misses a lot of detail in the fat below the brain area and around the eyes. ForkNet attempts to label many tissues and appears to be attempting to place them in the correct locations and order. Despite this, it has significant difficulty in that it labels a large portion of the head as background pixels. It particularly struggles with this in the front of the face, as tissues like the eye are completely missing. In addition, ForkNet places a large amount of muscle inside of the skull cavity. GRACE misses the eye in Test T1-MRI #3, but it is the only segmentation tool that correctly places the eye in Test T1-MRI #2. GRACE is particularly advantageous in its detailed segmentations of the eyes, cancellous and cortical bone, skin, fat, and muscle. It is comparable to HEADRECO in WM, GM, and CSF.

### 4.4 Improving reference segmentations

**Fig. 12.**
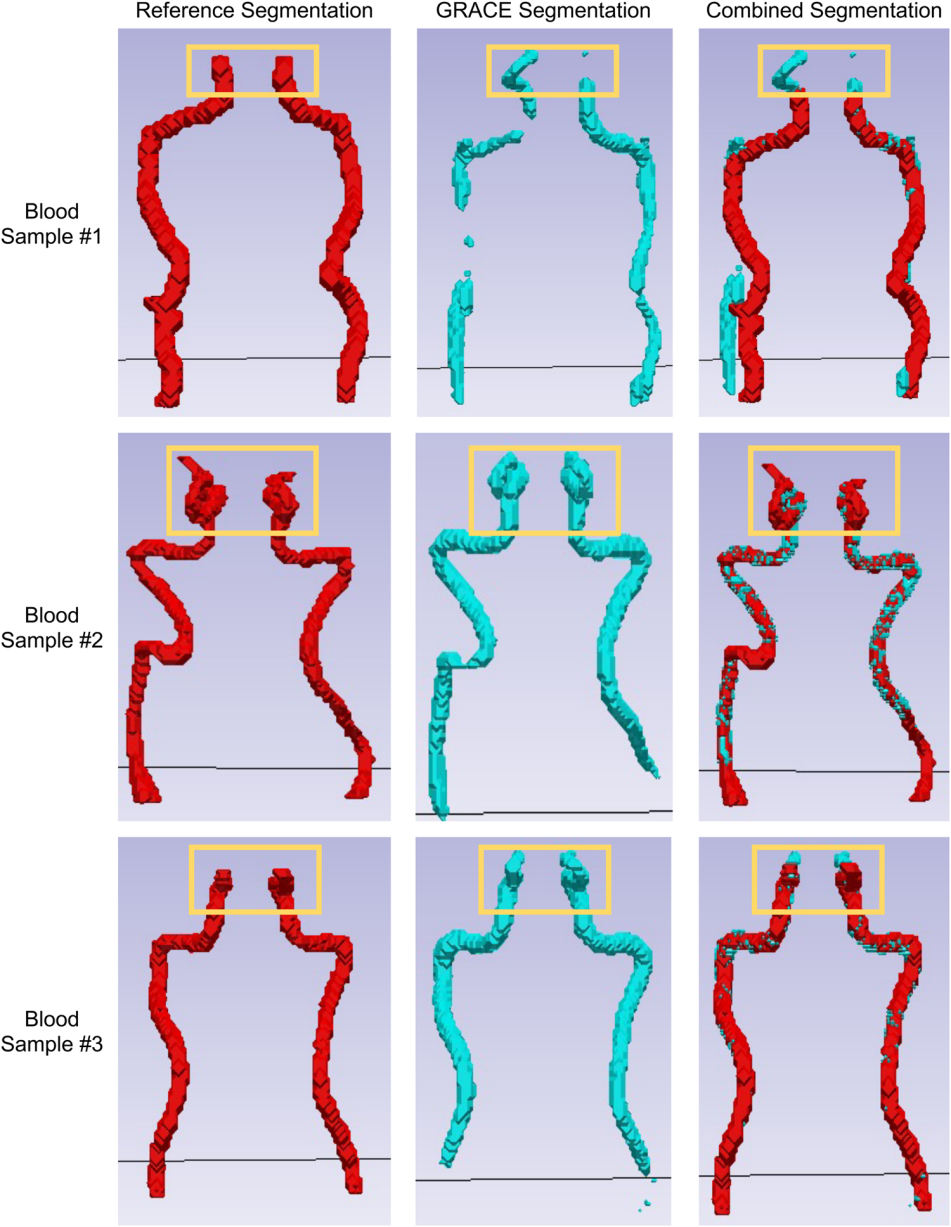
The blood samples from three T1-MRIs are shown here from the reference segmentation, GRACE segmentation, and combined segmentation. These figures show how GRACE blood segmentations may be combined with the reference segmentations to improve the final output. The highlighted region in the yellow boxes are areas where GRACE performs better in segmenting the blood, which can complement the reference segmentation.

Another strength of GRACE is its potential to improve the reference segmentations for challenging tissue types. Blood is one of the most challenging tissues to identify and label when the only imaging modality available is T1-MRI. Figure 12 shows the reference segmentations, GRACE segmentations, and combined segmentations for blood from three sample T1-MRIs. The preliminary qualitative results show that GRACE can improve the quality of the blood segmentation when paired with the reference segmentations. In some participants, the first run of GRACE segmentation produces a more accurate depiction of blood vessels by capturing the anterior portion of the artery that typically appears nearing the brain region. This particular region is difficult to distinguish with the human eye from the T1-MRIs. The reason for this difficulty is because the brightness and intensity of the blood vessel within the T1 starts shifting from dark/low to bright/high depending on the blood flow within these vessels at the time of MRI acquisition. After obtaining this information from GRACE, the segmentors correct all ground truth blood vessel labeling to ensure it would capture the most anterior portion. The correction is vital so that segmented vessels are consistent across participants. Once manual annotated vessels are corrected, GRACE is re-trained using the same dataset to improve its accuracy in capturing these vessels. These results focus on subjective assessments of improvement from the segmentors. Future works will quantify GRACE’s capabilities for improving semi-automatic segmentation in a human-in-the-loop fashion.

### 4.5 Segmentor consistency

**Fig. 13.**
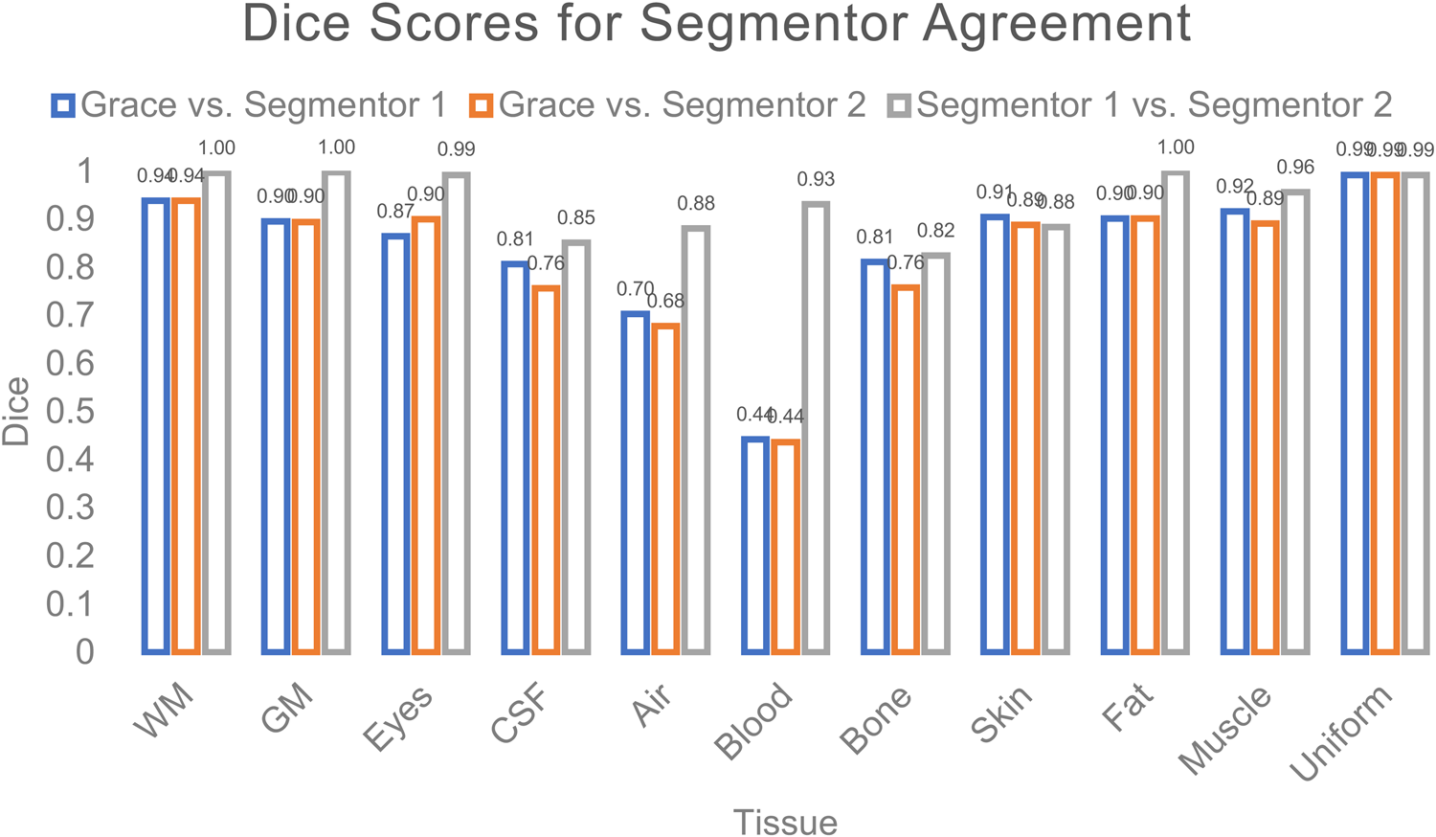
Bar chart featuring the Dice score versus tissue type. The best Dice score is 1 and the worst is 0. The three colors correspond to comparisons between different segmentor subgroups, and the data values are listed above each bar for convenience.

Figure 13 illustrates the Dice scores computed in practice head models. In the figure, blue bars correspond to GRACE’s agreement with manual segmentor 1, orange with GRACE’s agreement with manual segmentor 2, and grey with manual segmentor 1’s agreement with manual segmentor 2. All Dice scores for the manual segmentor overlap are between 0.79 and 0.99, which supports the consistency of the segmentors. As expected, the more complex tissue geometry and smaller number of voxels (e.g., blood vessels) yielded the lower end of percentage overlap between GRACE and the manual segmentors. GRACE is also consistent with the manual segmentors for the most part; however, future research could focus on improving in blood, fat, and muscle to be on par with the manual performance.

### 4.6 Comparison of the time computational cost across segmentation tools

Table 6 shows the average time that it takes to segment one T1 MRI volume using the segmentation tools that are featured in this work. The listed times are those required to segment the full raw 176 × 256 × 256 T1 MRI volume from one research participant into the maximum number of tissue types that are available from the corresponding segmentation tool. The estimated times represent the average time per participant across GRACE’s full testing dataset of 20 participants.

**Table 6.** This table shows the time that was required for each software to complete its whole-head segmentation.

Method	Number of head tissues^a^	Time for inference segmentation
SPM12	6	3 minutes
ROAST	6	30 minutes
MultiPrior	6	3 minutes
HEADRECO	8	1 hour
ForkNet	12	36 minutes
CHARM	10^b^	1 hour
GRACE	11	3-4 seconds

Note that this time is computed from the estimated segmentation time in the running logs for software that perform larger tasks (e.g., ROAST, CHARM, and HEADRECO all perform segmentation as part of NIBS pipelines. Only the raw segmentation time is listed here.).

a Number of Head Tissues does not include NIBS electrodes, since this is not a head tissue type.

b CHARM initially segments the 50 initial brain structures before producing the final 10 output tissues.

## 5 Discussion

In this work, we present a novel deep learning-based method for general, rapid, accurate, and comprehensive segmentation (GRACE) from a single volumetric head T1 MRI into 11 tissue types. GRACE compares favorably to six other popular and freely available software tools in a segmentation task of five major head tissue types. It achieves high segmentation accuracy over all 11 tissues. GRACE can attain higher precision at tissue boundaries than that of the traditional 3D U-Net architecture (Figs. 7 and 8). Specifically, GRACE segments tissue types that encompass both high percentages of the head volume (i.e., WM/GM/CSF) and smaller delicate anatomical structures (i.e., the lens in the eyes) with high fidelity. GRACE is an adaptable framework that can work for older populations and variable tissue types depending on the task. The current work supports GRACE’s ability to handle multiple tissue types through results on 5-tissue and 11-tissue tasks. This work demonstrates GRACE’s ability to serve as a fast and accurate head segmentation tool for older adult heads. Future work will study GRACE’s ability on other subject populations and tissue types.

GRACE achieves equal or better performance when compared to five freely available software tools and a traditional 3D U-Net on the 5-class task. Figures 9 and 10 show that GRACE compares favorably to CHARM, SPM, ForkNet, MultiPrior, and U-Net on all tissue types in the 5-tissue task. HEADRECO performs similarly to GRACE on GM and WM, whereas both algorithms achieve impressively high performance (Dice>0.90). One reason why HEADRECO performs well on the segmentation tasks could be because HEADRECO serves as the base input for semi-automatic segmentation in this paper. Therefore, there may be some inherent bias in the reference segmentations towards HEADRECO segmentation. The HEADRECO masks shown in these results are the HEADRECO raw outputs without manual correction. However, experienced segmentors manually correct significant issues in the HEADRECO masks before generating the final reference segmentations. These corrections also include additions that are not segmented by HEADRECO, such as the spinal cord, optic nerve, and brain stem. The segmentations that FreeSurfer and CHARM use are from participants between the ages of 20-50, which are significantly younger than the age range in this work. CHARM still has some specific issues in this older adult dataset outside of the base segmentation differences. For instance, CHARM underestimates the CSF volume across the testing dataset (see Fig. 11). Some of CHARM’s overall issues may have occurred because it was originally trained with young adult data. This work found that CHARM may perform suboptimal on older adult heads with the default parameters used within its command line functionality in SimNibs. These base results were improved by re-running CHARM with better registration parameters. Nevertheless, we found that CHARM’s performance was suboptimal when applied to the head models derived from older adults’ MRI compared to those from young adults. This appears to particularly be the case when only T1 MRIs are available. Therefore, we consider GRACE to be a competitive tool for segmenting the heads of older adults, especially with limited input modalities. The code for training ForkNet (including the model architecture) is available for only two tissues (WM and GM). The evaluation code and pretrained model are available for 12 tissues (Rashed et al., 2019, 2023). *This model was trained on data from younger adults (mean age: 43 years). The SPM outputs show decently high Dice scores but inconsistent Hausdorff Distances. One reason for this may be due to the “tissue isles,” or tissue placed as “dots” in roughly the right place but without proper connection. MultiPrior tool is promising in Dice score and Hausdorff Distance, but it falls slightly below the performance of tools like GRACE, HEADRECO, and U-Net. Overall, GRACE outperforms the other methods for this older adult dataset.*

The different segmentation tools that are featured in this work apply different assumptions during segmentation that may have impacted their performance. For example, CHARM is based on FreeSurfer segmentation, ForkNet is based on region growing and thresholding followed by manual correction, and HEADRECO is based on SPM and CAT12. Further studies would be necessary to exactly quantify what impact the choice of base segmentations has on the segmentor review and editing. Many of the methods in this paper are based on SPM-base segmentation methods, including HEADRECO, the MultiPrior tool, SPM, and our reference segmentations. GRACE can also be thought of as derivative of SPM due to the initiation of the reference segmentations. CHARM and ForkNet are not based around SPM segmentations; therefore, direct comparison to our reference segmentations is challenging. Indeed, HEADRECO makes certain assumptions that could propagate to our baseline segmentations. For instance, the coronal slice in Figure 3 shows that HEADRECO may underestimate the parcellation of brain regions such as the hippocampus, leading to an apparent increase in the volume of inferior CSF. On the other hand, CHARM does show some areas that may have possibly been more informative than the current data available. One example of this is that the blood compartment from CHARM includes the venous structures. In addition, the reference segmentations in this work follow the HEADRECO convention of closing the bone compartment structure around the spine. Evidence suggests that including only the tissues within a specific region between the electrodes and the brain target region may enhance modeling efficiency in tDCS (Wagner et al., 2013). Prior work has investigated the effect of reducing the head model coverage on the resulting electrical current difference in different target brain regions (Indahlastari et al., 2016). This previous investigation compared a head model spanning from the head apex down to the C3 vertebra to “truncated” head models: the most truncated model spanned from the head apex to the superior cerebellum (Indahlastari et al., 2016). The overall results indicated that even the most truncated models produced at most a 10% difference in the current density in target structures (Indahlastari et al., 2016). In addition, the GRACE dataset was constructed with a semi-automated segmentation approach followed by manual correction, which took between 20-30 hours per head for a total of 177 heads. Therefore, we believe that our approximation in the spinal region was reasonable given the extent of resources that were required to achieve large, curated dataset. The bone approximation may be reasonable for tDCS applications, but it could have had an impact on the performance metrics between toolboxes. For instance, CHARM segments the spine as separate structures rather than a closed shape. The difference in segmentation methods may have caused CHARM to quantitatively appear lower in terms of performance on the bone compartment.

Important advantages of GRACE also include its rapid processing speed and its ability to assist in semi-automatic segmentation. Performing automatic segmentation of one 3D head volume into 5-10 tissue types using existing freely available software tools ranges between 3 minutes and 1 hour. Further, many of the whole head segmentation software are within larger NIBS toolboxes. These toolboxes are typically designed to execute current flow modeling from T1 MRIs to produce electric field in one consecutive run. Obtaining only the segmented volume as an interim step within this pipeline may result in increased running time and prevent the ability of batch processing. GRACE’s current purpose would include providing improved segmentations within the larger NIBS toolbox. In addition, it could be used in other head modeling and segmentation tasks. The commitment for accurate segmentations could get very time and resource consuming. Eleven-tissue semi-automatic segmentation involving human segmentors takes about 20-30 hours. This process produces the most accurate segmentations; however, the time and personnel costs can be expensive. Purely automatic results produce faster results, but they may struggle to segment critical tissues at high accuracy from only a T1-MRI image. The authors acknowledge that the segmentations from non-fat-suppressed T1-weighted MRI scans may be limited by fat-shift artifact. However, many practical situations may not necessarily have all head modalities available (e.g., Computed Tomography for bone segmentation). Tissues like blood matter and cancellous bone are particularly challenging due to low contrast in MRIs (Rashed et al., 2020). Obtaining other imaging modalities improves segmentation results at the cost of increased impact on the research participants. Alternatively, leaving certain tissues out could neglect key differences captured in resulting electric fields due to the role of tissue conductivity (R. J. Sadleir et al., 2010). A major purpose of this paper is to introduce a tool that can segment the head as well as possible from only T1 inputs. This is highly applicable to cases where many other imaging modalities are not simultaneously available. Our manual segmentation team is extensively trained in how to segment the different tissue types, and segmentations are systemically quality controlled. As such, GRACE takes about 3 seconds for 11 tissue types and achieves close-to-human performance using only a T1-MRI. NIBS research can highly benefit from a tool that segments a large number of tissues from a single T1 volume (McCann et al., 2019; Nasimova & Huang, 2022; Pancholi & Dave, 2022; Puonti et al., 2020).

The rapid and accurate head tissue segmentation provided by GRACE could also help expedite semi-automatic segmentation with manual correction. For instance, GRACE outputs could replace certain stages of the reference segmentations in Figure 1. GRACE will be especially helpful for classes that are difficult for freely available fully automatic tools. For instance, air is a major struggle for tools like HEADRECO in our data from older adult heads. Other tissues like blood are missing from many segmentation toolboxes entirely. GRACE’s blood mask can capture certain regions in the blood which were originally missed or incorrect in some reference segmentations. The main region that is impacted from this is the hooked like structure in the “top” of the 2D blood Z-slice (Fig. 12). GRACE’s contribution to the blood masks allows the semi-automatic annotators to use GRACE masks to further improve the reference segmentations. Future works will explore this concept and quantify the usefulness of GRACE in improving semi-automatic segmentations.

GRACE can easily segment different numbers of tissues and adapt to smaller datasets. The deep learning backbone in GRACE enables flexibility and extendibility when it comes to diverse and novel tissue types; this is important for tasks where more tissue specificity improves the accuracy of treatment approximations. In this work, GRACE uses 11 tissue types based on previous works that show the effectiveness of these tissues in parameter stimulation for non-invasive brain stimulation such as tDCS (Indahlastari et al., 2021; Kasinadhuni et al., 2017; R. Sadleir et al., 2012). The experiment in which GRACE’s performance is compared on the 5-tissue task does not involve retraining. What this means is that the model is exclusively trained on 11 tissues and only fit to 5 tissues during post-processing. In addition to flexibility in the number of tissue types, the trained GRACE model can be adapted to different subject groups via transfer learning. The complete dataset includes 177 images in which 113 come from one scanner and 64 come from a different scanner. The 20-volume validation and testing sets (40 volumes between the two) are both evenly split into 10 images per scanner. The training set is 93 images from one scanner and 44 from the other scanner. Both scanners are comparable in testing performance in GRACE. Augmentation procedures like image rotations and Gaussian noise additions also help increase the model’s robustness to variability in inference data. The pre-trained model provided by this work may be able to serve as a basis for further finetuning on a smaller dataset from a different population. To the best of our knowledge, GRACE benefits from the largest dataset of manually corrected whole-head tissue segmentations of any full-head segmentation tool (177 volumetric T1-MRIs with manually corrected segmentations for 11 tissues). Future work will investigate the performance of GRACE on novel datasets via direct inference, transfer learning, and completely new training using randomly initialized weights (Kermany et al., 2018).

Comprehensive segmentation of diverse tissue types is another key contribution of GRACE. GRACE is promising to provide more accurate T1 MRI segmentation results for estimating parameters in transcranial electrical stimulation (TES) given the more accurate segmentation. One novel improvement of GRACE over existing tools is its ability to distinguish between fat tissue and general muscle tissue. Prior works find that the inclusion of fat content can impact electrical current estimations by up to 60% (Truong et al., 2013). Hence, the current work provides separate tissue definitions for muscle and fat. Another important inclusion is GRACE’s ability to separate bone tissue into cancellous (spongy) and cortical (compact) bone. Cancellous bone, which is more prevalent in older adults (Indahlastari et al., 2020), is more conductive than cortical bone. Hence, separating these two tissue compartments can particularly help in treatment planning for older individuals. Another distinction that GRACE makes is separating the eye compartment into aqueous vitreous (CSF) and lens, sclera (eye) components, which is a more accurate depiction of the human eye anatomy (Snell & Lemp, 2013). More importantly, including the correct compartment of aqueous vitreous is particularly important since the liquid (aqueous) is more conductive than a soft tissue compartment. Hence, labeling an entire eyeball as a single mask is not an effective representation of correct human anatomy in TES. To the best of our knowledge, GRACE is the first work in automatic head segmentation that make this distinction in eye compartments.

A common challenge across head segmentation software tools is to correctly segment blood compartments. GRACE found blood to be the most challenging among the 11 tissue types. Similarly, other algorithms did not include blood at all (i.e., HEADRECO, ROAST). This study found that the tissue that the trained human segmentors mark as blood encompasses different intensity ranges on the vessel exterior versus the vessel interior. In addition, blood was identified by our manual segmentors in less than 1% of the voxels in our 3D T1-MRI volumes based on the image contrast. This can be addressed by brain scans focused on measuring blood (i.e., arteriogram/venogram) to increase the accuracy of blood compared to using only structural MRI. However, a strength of GRACE is its ability to produce reasonable results with only T1-MRI. This is useful because collecting multiple imaging modalities from each participant can be challenging, infeasible, and expensive for NIBS and other applications.

An important future direction for this work will be to study the performance of GRACE in younger adult T1 MRIs. This extension will be important in validating the generalizability of the GRACE approach. Further, we plan to incorporate trustworthy machine learning into GRACE. Specifically, future work will estimate the uncertainties of prediction on tissue boundaries. The uncertainty measurements can integrate into deep learning models to improve their performance, calibration, and generalizability to out-of-distribution data. Another future direction is to use GRACE’s segmentation to improve FEM and electrical current estimation for non-invasive brain stimulation. GRACE has the potential to enable personalized stimulation using its rapid and accurate head tissue segmentation and to address the heterogenous responses in NIBS and related clinical applications.

## 6 Conclusion

In summary, we present a new method (GRACE) for automatic segmentation of 11 different head tissues from T1-MRI scans. GRACE is trained and validated on the largest database of whole-head tissue segmentation with high fidelity reference segmentations from T1 MRIs (n = 177). GRACE compares favorably to six other freely available tools (CHARM, HEADRECO, SPM, U-Net) in simplified segmentation tasks of the seven and five major head tissue classes. GRACE achieves relatively high accuracy in conventionally challenging tissues, including those associated with an older adult cohort (e.g., brain atrophy and osteoporosis). Compared to lengthy segmentation using existing software tools, GRACE only takes approximately 3 seconds to segment a volumetric T1 MRI. The deep learning backbone architecture offers flexibility and extensibility to novel tasks and different populations with smaller dataset size. GRACE currently segments 11 tissues in T1 MRIs; however, it can be generalized to different tissue labels and imaging modalities as needed, which will be a future direction of this work. GRACE’s accuracy, speed, and tissue flexibility provide abundant opportunities for downstream tasks. Currently, GRACE is a very useful tool for comprehensive and accurate segmentation in older adult heads. This performance will be useful in partnership with tools that perform downstream tasks in head modeling pipelines for precision modeling in cognitive aging and dementias.

## Data Availability

The data analyzed in this study are subject to the following licenses/restrictions: data are managed under the data sharing agreement established with NIA and the parent R01 clinical trial Data Safety and Monitoring Board in the context of an ongoing Phase III clinical trial (ACT study, R01AG054077). All trial data will be made publicly available 2 years after completion of the parent clinical trial, per NIA and DSMB agreement. Requests for baseline data can be submitted to the ACT Publication and Presentation (P&P) Committee and will require submission of a data use, authorship, and analytic plan for review by the P&P committee (ajwoods@phhp.ufl.edu). Requests to access these datasets should be directed to ajwoods@ufl.edu. Code is available at https://github.com/lab-smile/GRACE.
